# Analyzing historical land use changes using a Historical Land Use Reconstruction Model: a case study in Zhenlai County, northeastern China

**DOI:** 10.1038/srep41275

**Published:** 2017-01-30

**Authors:** Yuanyuan Yang, Shuwen Zhang, Yansui Liu, Xiaoshi Xing, Alex de Sherbinin

**Affiliations:** 1 Institute of Geographic Sciences and Natural Resources, Chinese Academy of Sciences, Beijing 100101, China; 2 College of Resources Science and Technology, Beijing Normal University, Beijing 100875, China; 3 Northeast Institute of Geography and Agroecology, Chinese Academy Sciences, Changchun 130102, China; 4 Center for International Earth Science Information Network (CIESIN), Earth Institute, Columbia University, Palisades, NY 10964, USA

## Abstract

Historical land use information is essential to understanding the impact of anthropogenic modification of land use/cover on the temporal dynamics of environmental and ecological issues. However, due to a lack of spatial explicitness, complete thematic details and the conversion types for historical land use changes, the majority of historical land use reconstructions do not sufficiently meet the requirements for an adequate model. Considering these shortcomings, we explored the possibility of constructing a spatially-explicit modeling framework (HLURM: Historical Land Use Reconstruction Model). Then a three-map comparison method was adopted to validate the projected reconstruction map. The reconstruction suggested that the HLURM model performed well in the spatial reconstruction of various land-use categories, and had a higher figure of merit (48.19%) than models used in other case studies. The largest land use/cover type in the study area was determined to be grassland, followed by arable land and wetland. Using the three-map comparison, we noticed that the major discrepancies in land use changes among the three maps were as a result of inconsistencies in the classification of land-use categories during the study period, rather than as a result of the simulation model.

Decadal-centennial land use and land cover change (LUCC) has been considered as a vital driver of global environmental change^1^,^2^,^3^,^4^,^5^. Historical land use information is essential in understanding how anthropogenic land use and land cover change has influenced the temporal dynamics of environmental and ecological issues, including soil degradation^6^,^7^, water quality^8^,^9^, habitat loss and fragmentation^10^, biodiversity loss^11^,^12^, climate change^13^,^14^,^15^ and, most importantly, the global carbon balance^16^. However, a primary obstacle in assessing the consequences of land use changes is the lack of high-resolution, spatially explicit and thematically complete historical land-use change data, as well as the conversion types that feed into the models related to the above ecological issues^5^.

Due to a lack of available adequate historical datasets, the reconstruction of historical land use/cover relies on existing databases containing local/regional level statistics or records, population statistics, historical maps and model assumptions^5^. Recently, substantial progress has been made in gathering historical land use data and producing historical reconstructions, with an increase in investigations at both the global and regional scales^17^,^18^,^19^,^20^,^21^,^22^,^23^,^24^,^25^,^26^,^27^,^28^,^29^,^30^,^31^,^32^. The changes have been discussed in detail through a review of historical reconstruction methods of LUCC^33^. The majority of current studies fail to thematically represent the land area in its entirety, and also fail to include competing land-use categories and land conversion types^5^. For example, present researches target arable land, wetland and forestland, but do not provide information regarding land-use categories such as settlements, water bodies or other land use types.

Model-based reconstructions of land-use change are rapidly advancing as anthropogenic land-use change is one of the most important drivers of environmental change. As the accumulation of human activities’ impact on the earth’s surface, present land use pattern contains the essential information about historical land use patterns. Meanwhile, remote-sensing technique makes it feasible and convenient to observe the global characterization of land use/cover with high resolution and thus the present land-use spatial pattern is the most crucial information for historical land reconstruction. In general, the spatial allocation method is under the assumption of similarities between historical land use spatial patterns and present spatial patterns. Related models have been divided into three types according to the various effects on controlling the similarities: totally dependent, partially dependent and dynamically dependent^34^. The differences among the three types lie in the different spatial allocation principles. The totally dependent approach means that historical land-use pattern is completely decided by the present one in the reconstruction model. That is, the proportion of the allocated area (amount) in each grid accounting for the total area (amount) in an administrative area (country or province) is an invariable in spatial allocation by adopting downscaling or other ways. The partially dependent approach is to avoid “using today’s pattern for historical reference” and it focuses on the driving mechanism research of spatial pattern. Generally, the partially dependent approach is based on an assumption that the range of historical agricultural activities could not exceed the present one. The dynamically dependent approach has the sophisticated algorithms and takes the effect of time into consideration^34^, initially proposed by Goldewijk^35^ in his HYDE 3.1 (History Database of the Global Environment) application^35^. The driving force of land use process has to be clarified and the land-use change trajectories have to be analyzed before using the partially dependent approach. After this, a land use forecasting model (e.g., cellular automata, Geomod) as a carrier can be used to produce a backward simulation. In this context, to enhance our understanding of the degree and extent of global and regional anthropogenic changes in land use patterns^36^,^37^, a spatial-explicit modeling framework (HLURM: Historical Land Use Reconstruction Model) is explored and proposed in this paper. Currently, there is a growing demand for harmonized, spatially explicit and high resolution land-use change products. Our model will aim to satisfy this demand. This model approach can also produce backward projections by analyzing land use changes over time, and it is a dynamically dependent approach based on the assumption that current spatial land use pattern is intrinsically linked to historical patterns.

As the third largest and most populated country, China has a long history of agricultural civilization and its land use has undergone great changes owing to significant transformation caused by human activities, natural factors and their impact on the climate and environment^38^,^39^, which plays a significant role in the global LUCC pattern^40^. Over the last century, northeastern China has experienced large-scale population reflux of Chuang Guandong migration and major land reclamation process influenced by lifting the ban on the Qing Government’s prohibition policy^41^,^42^,^43^. As a result, it is one of the regions in China witnessing dramatic LUCC in a century time scale^41^. Here, considering the richness of historical documents and the availability of regional LUCC data derived from the historical topographic maps, physical environmental background maps including those of terrain, climate, geology, soil, vegetation and hydrology as well as socioeconomic statistical data based on our previous research, we choose to represent Zhenlai County, located in northwestern Jilin province, as a case study. As a part of farming-pastoral ecotone of northern China, the eco-environment in Zhenlai County is very fragile and is liable to the disturbance of various factors^41^. In this research, we try to explore a spatial-explicit modeling framework (HLURM) by using the dynamically dependent spatial-allocation approach and then apply this modeling framework to the historical reconstruction of spatial distribution of land use/cover in the early reclaimed time of northeastern China to check its modeling behavior. The overall objectives of this paper are: (1) to reconstruct historical land use during the early period of reclamation (1930 s) in Zhenlai County using a 90 m × 90 m HLURM model to generate a backward projection; and (2) to validate the results of the reconstruction by means of a three-map comparison, assessing its accuracy by classifying pixels into the following four types: null successes, hits, misses and false alarms. This research will make us understand better the historical land use and land cover change over the past century in northeastern China and provide harmonized, spatially explicit and high resolution land use change products to ecological issues’ study.

## Materials and Methods

### Study Area

**Figure 1:**
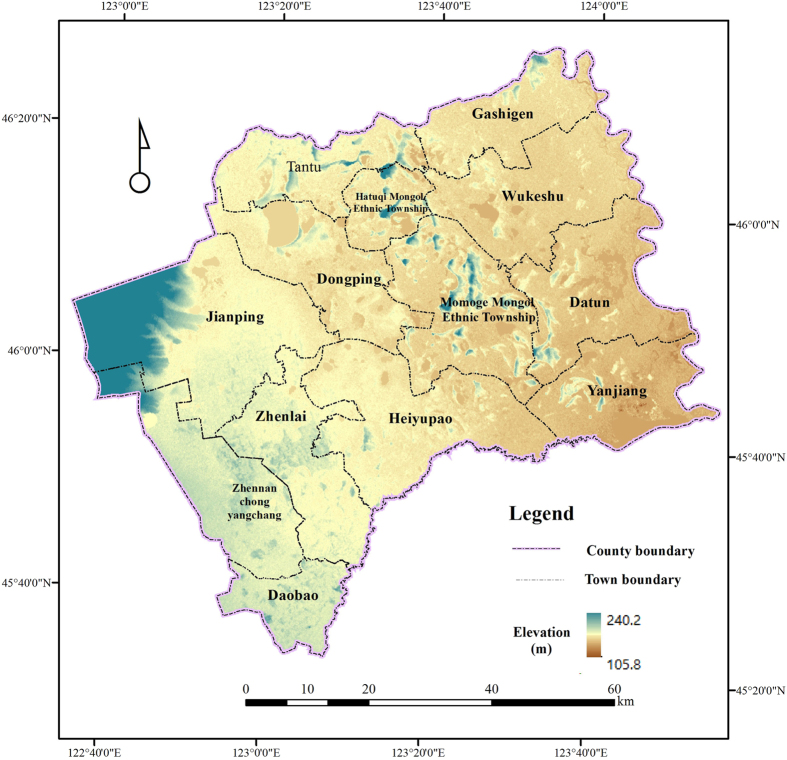
Map of the study area, Zhenlai County (map created using ARCGIS 10).

Zhenlai County (N45°28′–N46°18′, E122°47′–E124°04′; Fig. 1) is a typical part of the farming-pastoral ecotone of northern China. Under the governance of Baicheng city, Zhenlai County lies in the northernmost part of Jilin province, and it borders Heilongjiang to the east and Inner Mongolia to the west. Historically, the region comprised nomadic land for Mongol princes, and it was only after the enactment of a policy lifting the ban on land reclamation during the late Qing dynasty (1902) that inhabitants were able to reclaim land. In 1910, the county was established as Zhendong County and was later merged with Laibei County in 1947, and this combined region was later renamed Zhenlai County. The region has experienced dramatic LUCC over the past century, and the land reclamation process is relatively complete. The county is rich in geomorphologic features with high elevation in the northwest and low elevation in the southeast. The northwestern area is adjacent to the Greater Hinggan Range and the central areas mostly comprise rolling hilly land. The eastern and southern regions surround the Nenjiang and Tao’er Rivers, respectively, forming a fertile flood plain on both riverbanks. The main soil types include chernozem, alluvium soil, alkali soil and meadow soil. As this region is located in the inland mid-latitude areas, it comprises a temperate, continental monsoon climate with distinct seasonality. Mean annual rainfall is 402.4mm and is unevenly distributed across time. Mean annual evaporation is 1755.9 mm, which is approximately four times greater than the mean annual rainfall. This low volume of precipitation and high level of evaporation results in a drought-prone climate in the area, especially in the spring. The mean annual temperature is approximately 4.9 °C.

### Data

One Landsat MSS (Multispectral Scanner) and two Landsat TM (Thematic Mapper) images were selected for the years 1976, 2000 and 2005. These remote sensing images were used to interpret and vectorize land use data obtained from the United States Geological Survey (USGS; http://glovis.usgs.gov/). A series of topographic maps from the 1930 s (in the year of 1932 drawn to scale at 1:50 000, in the year of 1935 at 1:100 000 and 1:500 000, respectively) were also collected and digitized. The historical spatiotemporal distribution of land use and land cover in 1954 was also reconstructed by consulting and incorporating information relating to terrain, climate, geology, soil, vegetation, hydrology and socioeconomic statistical data from topographic maps and physical environmental background maps^44^,^45^. Soil data was digitized from the “*Local Record of Zhenlai County*”. A slope raster was generated using a 90 m raster DEM obtained from the Shuttle Radar Topography Mission (SRTM).

### Classification system

As land use types indicated on the topographic maps and remote sensing images used in this study differed, we initially produced a map series with unified contents. In order to make temporal comparisons, the maps also had to be thematically generalized. Considering both the local characteristics as well as the predominant land use classification system used in China^46^, the available land classes were aggregated into seven suitable land-use categories for this study: arable land, forestland, grassland, water, settlement (urban and rural construction), wetland and other unused land (including sand, saline-alkali soils and bare land)^47^,^48^.

### HLURM model

The Historical Land Use Reconstruction Model (HLURM) provides a high-resolution reconstruction of historical land use spatial distributions. The model outputs are based on the assumption that the present spatial pattern of land use is intrinsically dependent on a historical pattern.

#### HLURM theory

Considering the natural, social and economic predictors of changes in land use and land cover, HLURM takes into account regional land use changes since the advent of remote sensing. This was undertaken by taking the grid as the research unit, calculating the probability of each land type in each grid to determine the most likely land-use category and then using the land type with the largest probability to reveal the land use in the research units. A cellular automata (CA) model was then used to produce a backward simulation as a carrier.

#### HLURM modeling framework

**Figure 2:**
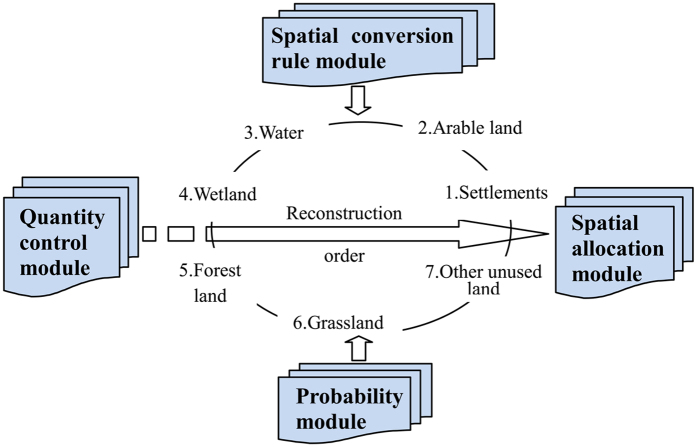
HLURM’s four modules.

The HLURM model consists of four main modules: quantity control module (QM), spatial conversion rule module (CM), probability module (PM) and spatial allocation module (AM) (Fig. 2).

Quantity control module (QM), also known as the demand-constraint module, is the basis of HLURM model and also the premise of the spatial reconstruction. This module can be operated and run independently of the HLURM model. In general, total quantity of land use can be determined in two ways: (a) collecting historical documents and calibrating the available data; and (b) modeling quantitatively from the simple historical trend extrapolation method to the relatively complicated economic model. Appropriate model of backward projection for land use quantity should be chosen according to the actual circumstance.

Spatial conversion rule module (CM) establishes historical rules for land use and land cover change. This module contains analysis of both land use conversion sensitivity and land use conversion sequence. Land use conversion sensitivity monitors the conversion characteristics and trends among land-use categories during each time interval; obtains the transformation probabilities for land dynamics in the study period; and clarifies the trajectories of land use changes. These factors provide fundamental basis for analyzing the land use conversion sequence. CM will then reveal land dynamic characteristics and provide essential data for the following probability module, as well as producing the constraints for the spatial allocation module.

Probability module (PM) calculates the probability of each land type in each grid based on probability theory. By analyzing the relationships between both various natural environmental conditions and socio-economic conditions and land-use distributions, a land suitability evaluation map is produced to exhibit the possible spatial distribution of historical land cover. According to current research about land use and land cover change, three assumptions of HLURM are proposed to operate the PM module: 1) The current spatial land use pattern is intrinsically dependent on a historical pattern; 2) The boundary of historical land use with human activities, such as arable land and settlements, does not exceed the union range of each land use type in the study period; 3) The natural environmental factors of land suitability have not changed over time during the past century due to the limitation of data collection.

Spatial allocation module (AM) distributes the quantity of each land use into the geographical locations and spatially explicit outcomes according to certain principles or approaches. It then realizes the backward projection of historical land use. For specific spatial allocation, downscaling can be used in the descending order of distribution probability under the control of total land-cover area and other distribution factors.

According to the influence of human activities on land use and the characteristics of land use, the reconstruction order for land-use categories in this study is as follows: settlements, arable land, water, wetland, forestland, grassland and other unused land. Different spatial constrains are established for land-use categories and then spatial grid allocation is operated under these constrains.

### Three-map comparison

The maps used for the three-map validation of the LUCC model included reference time 1, reference time 2 and simulation time 2. Comparisons between the maps of reference time 1 and reference time 2 characterized the observed changes in the maps, reflecting the dynamics of the land use. Comparisons between the maps of reference time 1 and simulated time 2 characterized the predicted changes, demonstrating the model behavior. Model validation estimates the agreement (or error) stemming from the comparison between the maps of simulation time 2 and the reference (or real) time 2. Here, the model accuracy was assessed by determining the four components of accuracy and error, namely, null successes (accuracy of observed versus predicted persistence), hits (accuracy of observed versus predicted change) misses (error due to observed change predicted as persistence) and false alarms (error due to observed persistence predicted as change). The summary statistics for error due to quantity (EQ, Eq. 4) and error due to allocation (EA, Eq. 5) were also assessed^49^,^50^. Quantity error reflects the inability of the model to perfectly predict the quantity of net change, and is not influenced by spatial allocation. Conversely, allocation error is associated with the inadequate ability of the model to allocate pixels of change across the landscape. This error derives from the spatial allocation algorithm, which is associated with the independent variables in the model. The error might be sensitive to any modification of the spatial allocation algorithm. In addition, the figure of merit (FOM, Eq. 6) and the three ratio indices (Eqs 7,8,9) that quantify the amount of hits, misses and false alarms relative to the observed change, were also determined. The FOM is calculated by dividing the hits by the sum of hits, misses and false alarms and, in the case of the models that simulate several categories, removing the partial hits from the numerator. This measure allows a more realistic assessment of the cell-to-cell coincidence between simulated and real maps than more commonly used metrics, such as the kappa index or overall accuracy, which are usually calculated using the entire surface area^51^. The FOM ranges from 0% (no overlap between observed and predicted change) to 100% (perfect overlap between observed and predicted change)^52^.


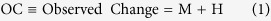



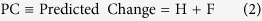



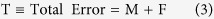



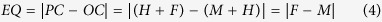



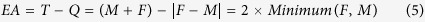



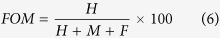



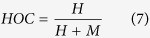



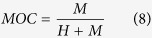



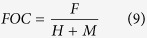


where, H, M and F are the hits, misses and false alarms; HOC, MOC and FOC are, respectively, the ratio of hits, misses and false alarms to the observed change, which is the summation of the hits and misses.

## Results

### Quantity control module

**Figure 3:**
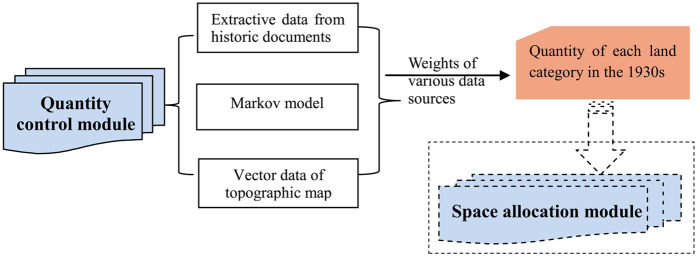
Quantity control module.

Based on available historical data, the total quantity of each land use was calculated according to the weights of various data sources, such as historical documents, Markov model results and vector data derived from topographic maps (Fig. 3).

The spatiotemporal Markov Chain model uses transitional probabilities to model change over time among land-use categories, and is able to simulate several land-use categories simultaneously. However, due to temporal changes in the various land-use categories, transition matrices describing various types of land cover from *t* *−* *1* to *t* might not be totally consistent with those in the period between *t* and *t* *+* *1*. Thus, the transition matrices were modified by analyzing land use changes over the last few decades, and were also used to define the rules for AM, assuming that current spatial pattern of land use is inherently dependent on the historical pattern. In this context, in order to account for the period between 1932 and 1954, transition matrices were revised between 1954 and 1976 according to the rules governing LUCC in the study area from 1954 to 2005. The analysis of land use changes indicated that nearby forests and wetlands would not be utilized until all of the grassland suitable for farming was reclaimed^47^. Hence, we added the grid numbers for wetland converted into arable land from 1954 to 1976 to grassland. In addition, climate change, especially precipitation, had a significant impact on wetlands due to the smooth landscape in the study area. Gao^53^ indicated that northeast China experienced a drier period between 1900–1935, a wetter period between 1936–1959 and another dry period after 1960. In light of this we adjusted the area of “wetland → water” into the area of “wetland → grassland” from 1954–1932. Finally, the area of each land use was obtained by modifying Markov results.

Weight is often used to develop a set of relative weights for a group of factors in a multi-criteria evaluation. Here, the analytic hierarchy process (AHP) approach, calculating from “WEIGHT-AHP weight derivation” module of the IDRISI Selva software, was used to determine the weights of land-use categories from different data sources in the 1930 s based on the area of various land use from multi-source data and the reaction characteristics of land-use categories in these different data sources. Under the control of 531606.14 ha of the total study area, the areas of land-use categories in the 1930 s were obtained. Table 1 shows that grassland dominated the largest area of land cover (334798.79 ha) with arable land (129966.07 ha) being the second most dominant. The areas of forest land, water, settlement, wetland and other unused land were 675.69 ha, 9274.14 ha, 3758.57 ha, 51514.18 ha and 618.69 ha, respectively.

### Spatial conversion rule module

**Figure 4:**
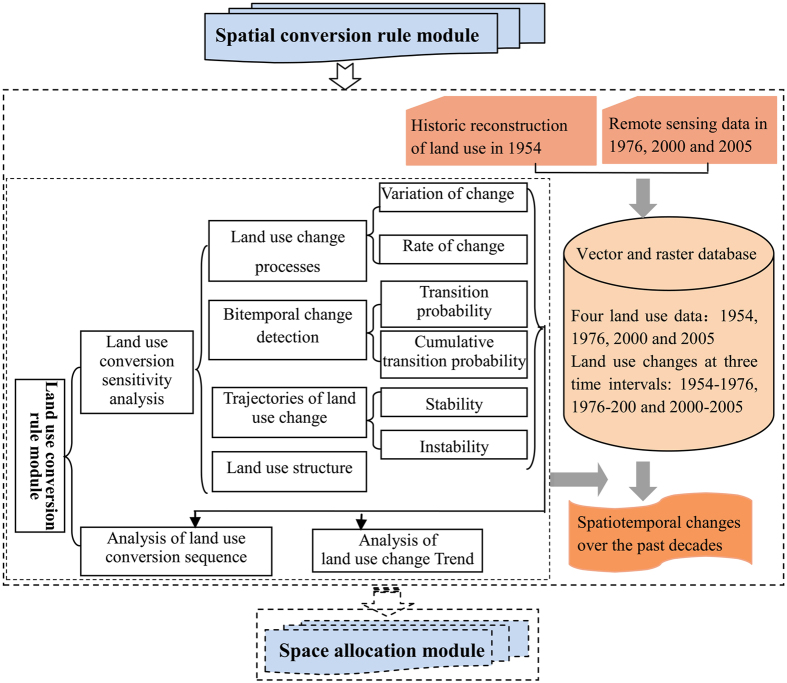
Spatial conversion rule module.

Research regarding the spatial conversion rule module was comprehensively explained and discussed in the spatiotemporal changes’ analysis from 1954 to 2005 in the study area^47^,^48^. Results from our analysis (Fig. 4) enable the following conclusions to be made according to spatial conversion rules for various land use: (1) Arable land expanded at the expense of grassland and wetland. At the same time, a large proportion of grassland was converted into unused land, reflecting the remarkable environmental degradation experienced in Zhenlai County during the study period. (2) Trajectory analysis of land use and land cover change demonstrated that settlements, arable land and water bodies remained relatively stable in terms of coverage and spatial distribution, while grassland, wetland and forestland demonstrated weak stability. (3) While natural processes dominated environmental change in the study area, anthropogenic influences also played an important role. (4) The Lorenz curve/Gini coefficient indicated that arable land was the most scattered, whereas forestland was the most concentrated.

### Probability module

**Figure 5:**
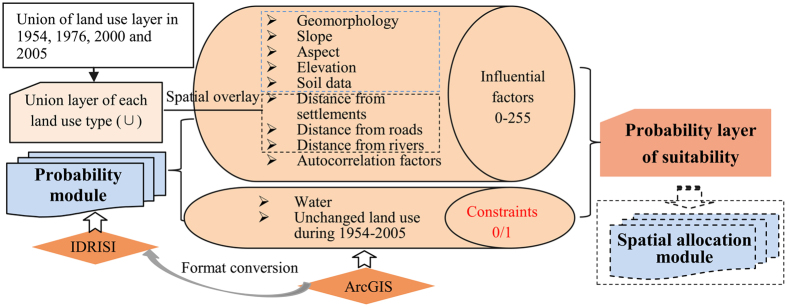
Probability module.

**Figure 6:**
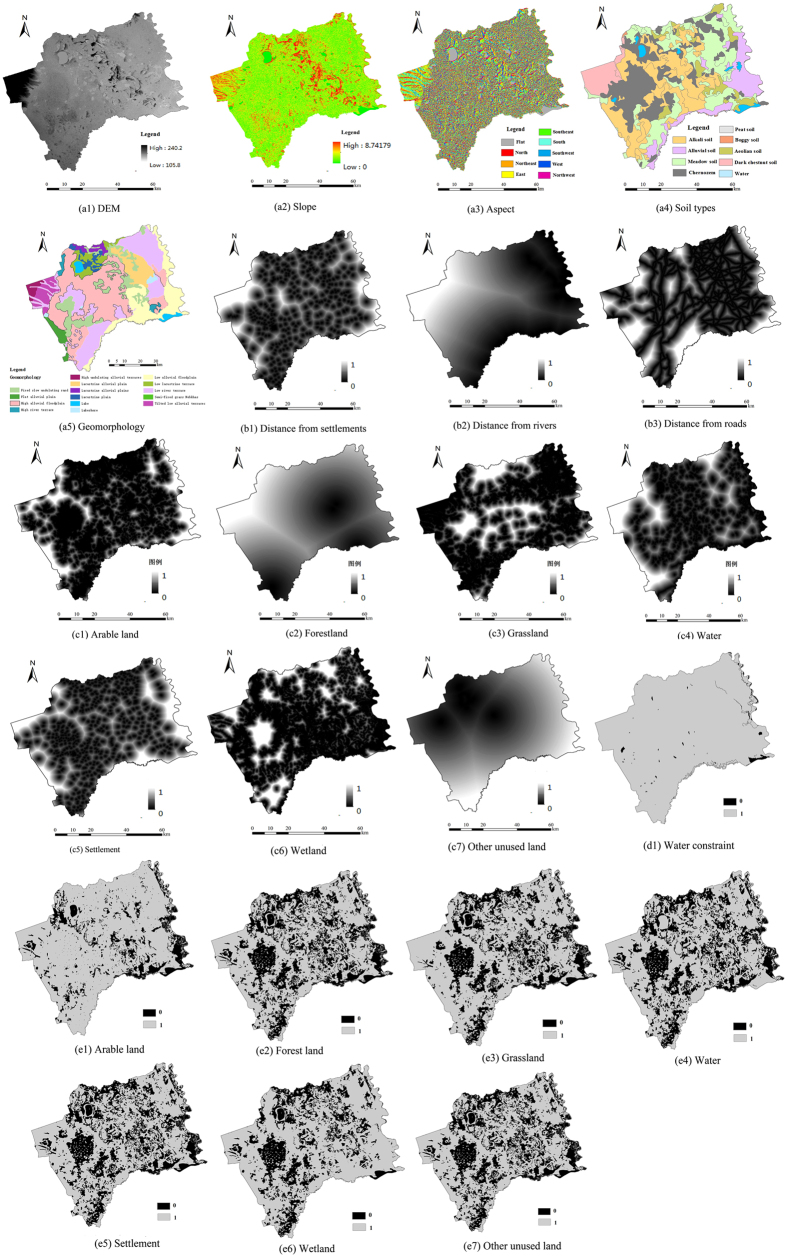
Environmental influential factors for suitability maps (**a1**–**a5**); human disturbance factors (**b1**–**b3**); spatial autocorrelation distance factors (**c1**–**c7**); and constraint images for water (**d1**) and unchanged land cover (**e1**–**e7**) (map created using ARCGIS 10).

Factors and constraints were the two types of criteria used in this study (Fig. 5); a factor signified a continuous degree of fuzzy membership (in the range of 0 ± 255), while constraints limited the alternatives altogether (i.e. fuzzy membership is either 0 or 1)^54^. The factors for suitability maps included the following three kinds: environmental influential factors (geomorphology data, topographic data such as slope, aspect and elevation, soil data, Fig. 6a); human disturbance factors (distance from settlements, rivers and roads, Fig. 6b); and autocorrelation factors (Fig. 6c). Each grid cell in all of the digital raster maps represented an area of 90 m × 90 m, which is considered an accurate representation of the land-cover in the study area. Such precision avoids losing the resolution of the data, though it could result in data redundancy. Aiming to union each land-use category by overlaying land use maps in several time points, this module calculated the percentage of each land-use category distributed in these five natural factors to represent the probability of each land use in each grid under different environment backgrounds by intersecting the above union (∪) dataset with various environment background data. We also digitized settlements, rivers and road layers from the 1930 s topographic maps, calculated their Euclidean distances and then dispersed the distance value within the range of 0–1; where 0 represented the nearest distance and 1 represented the farthest distance. In addition, in accordance with the principle of spatial autocorrelation that suggests that certain land use types favor a certain land cover^55^, we also used the spatial autocorrelation distance of each land-use category based on 1954 land use map. Finally, the hundred-mark system about the percentage of each land-use category in the five natural factors was converted into a 255-mark system while the distance values with the range of 0–1 about human disturbance factors and autocorrelation factors were reverse-extended to 255–0. These were then calculated by standard formula for grid computing to meet the demand of this module.

Here, two constraints were considered: water and unchanged land-cover during 1954 and 2005. Based on the previous three assumptions of the PM, we concluded that the unchanged land use from 1954–2005 had existed in 1932. Considering that the other six land cover types do not typically occur on bodies of water, water layers digitized from the 1930 s topographic maps (Fig. 6d1) were subjected to the same constraints as all the other land use/cover types, excepting themselves. In terms of long-term landscape development, the degree of unchanged land use across the study period was relatively stable and consistent in its spatial distribution. We thus made the assumption that the degree of unchanged land cover over the past 60 years had also existed in the 1930 s, and the present land use pattern is dependent upon the historical one. Thus, a particular land use type could not develop where a different land cover had existed at that time (Fig. 6e).

**Figure 7:**
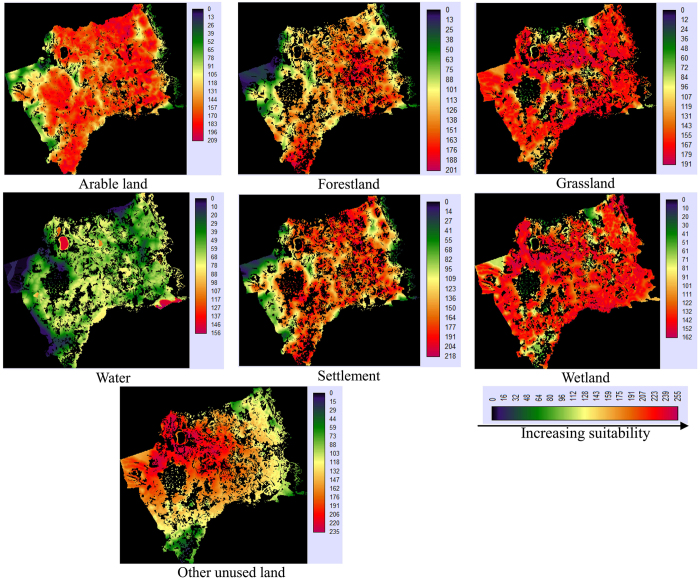
Suitability maps for various land-use categories (map created using IDRISI Selva).

We made use of the MCE process involving criteria of varying importance in accordance with decision makers along with information regarding the relative importance of the criteria. It is generally obtained by assigning a weight to each factor. The weights given to different factors in this study were generated using Saaty’s Analytical Hierarchy Process (AHP), where a larger weight denotes a more important criterion in terms of overall utility (Table 2). The probability module was completed using IDRISI Selva software and the suitability maps for various land-use categories are shown in Fig. 7.

### Spatial allocation module

**Figure 8:**
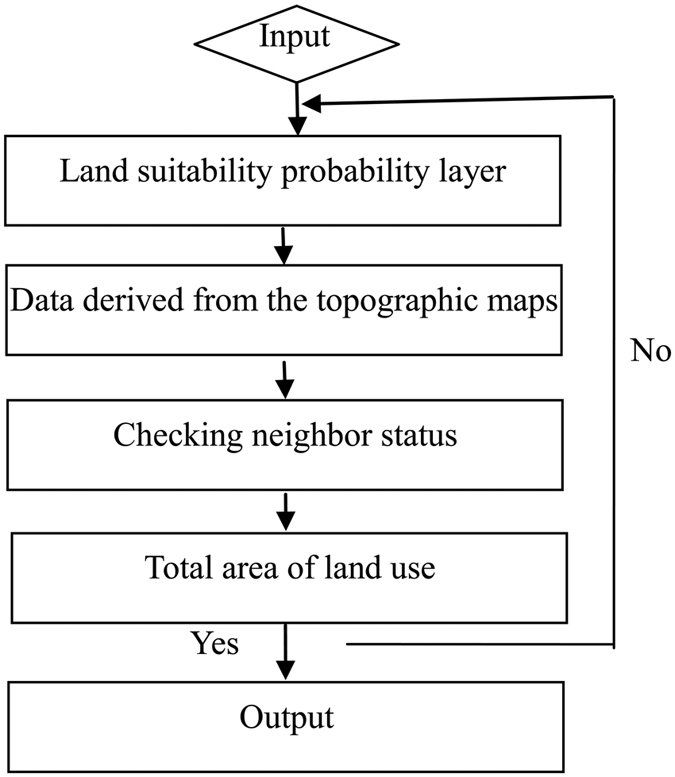
Flow chart of spatial allocation module.

This module, based on the algorithm of the cellular automata (CA) model, has two kinds of spatial constraints: (1) interpretation results of topographic maps; and (2) the spatial probability of each land-use category. The specific spatial allocation process is shown in Fig. 8. There were a number of procedures in this module: (1) whether or not the probability of land use is the largest in the land suitability probability layer; (2) whether or not it is consistent with the data derived from the topographic maps; (3) checking neighbor status to judge whether or not the number of one land-use category around the neighbor grids is equal to or greater than five; (4) whether or not this certain land use type reaches the upper limit of its total quantity. If the land use type has reached it upper limit then the procedure ends; otherwise, the procedure returns to the above process to continue to judge; (5) the reconstruction order for land-use categories is as follows in the process of spatial allocation: settlements, arable land, water, wetland, forestland, grassland and other unused land.

**Figure 9:**
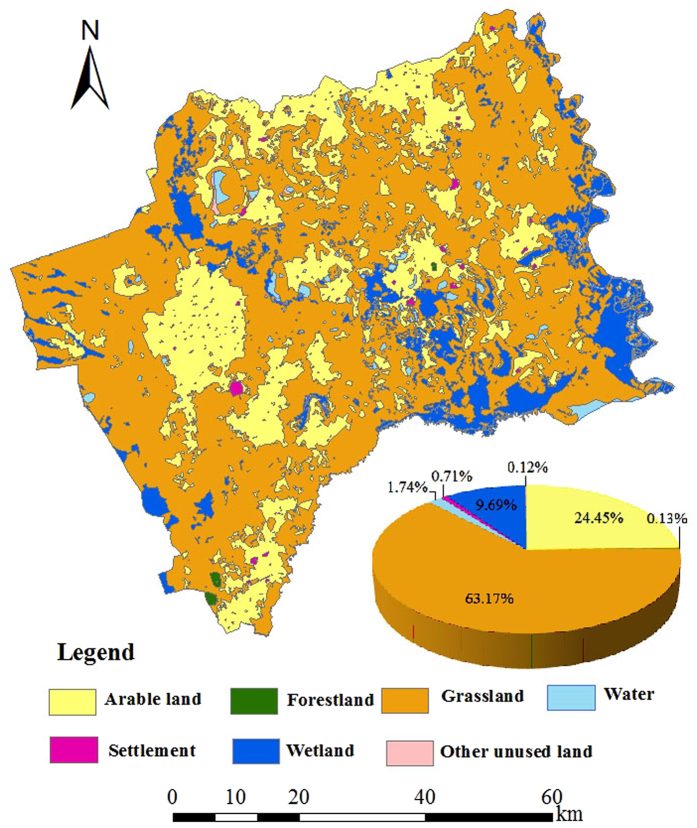
HLURM simulation of land-use map in the 1930 s (map created using Matlab R2012b).

The spatial allocation module is programmed in Matlab software and the 1930 s land-use map simulated by the HLURM model could be output by spatial allocation module (Fig. 9). It can be seen that grassland dominated land cover (63.17%), a result which was expected given the large number of immigrants that settled in this area, resulting in the reclamation of wildland and grassland. Anthropogenic activities intensified following the enactment of the policy lifting the ban on land reclamation in northeastern China in the late Qing Dynasty. Arable land accounted for the second largest land cover (24.45%), the majority of this land type being located in the flat areas of the central and western parts, as well as in the north. The arable land was also found to be scattered between the other areas. The wetland accounted for 9.69% of the total area and it was mainly distributing to the east and south along the Nenjiang River and the Tao’er River. The area of water bodies consisted of 1.74% of land cover while the area proportions of settlements, forestland and other unused land were 0.71%, 0.13% and 0.12%, respectively.

## Discussion

Through producing a backward projection, the HLURM model creates a high-resolution historical reconstruction of land use and land cover in Zhenlai County in the 1930 s. A common method to test the reliability of historical reconstructions involves comparing with information from a variety of independent sources^56^. Detailed historical maps are valuable sources for land cover reconstruction as these can be used to directly digitize historical land cover. We compared the contemporary HLURM model results with historical 1:100,000 topographic maps as these allowed us to analyze land use on a regional scale^57^. According to a three-map comparison, we were able to validate the model by identifying all potential prediction successes and errors. The three maps included the observed 1954 land-use map, the observed 1932 land-use map digitized from the topographic maps and the predicted 1932 land-use map. As six land-use categories were present in the digitized topographic maps, we decided to combine wetlands and other unused land types into unused land in both the reference 1954 map and simulated 1932 map. Thus, six land categories were defined for comparison: arable land, forestland, grassland, water bodies, settlements and unused land.

**Figure 10:**
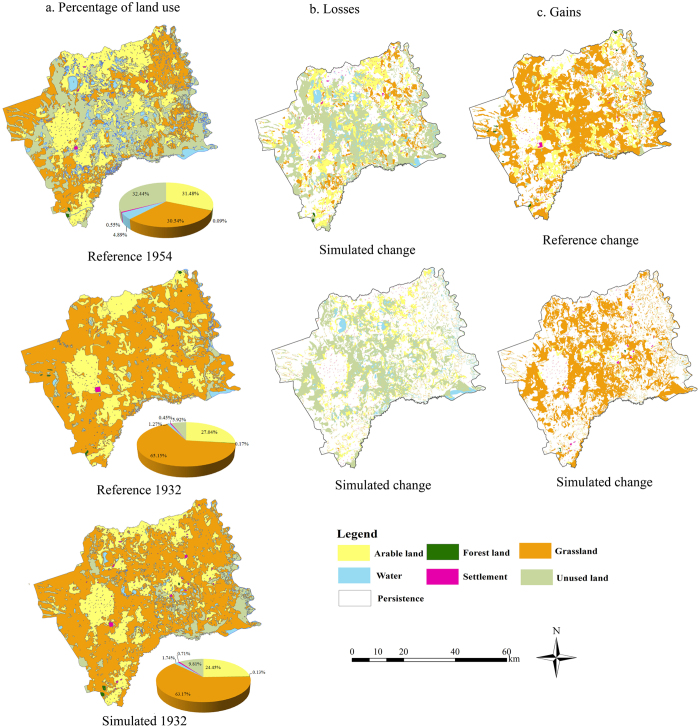
Percentage of each land-use category at three time points and the changes at two time intervals in the study area (map created using ARCGIS 10).

**Figure 11:**
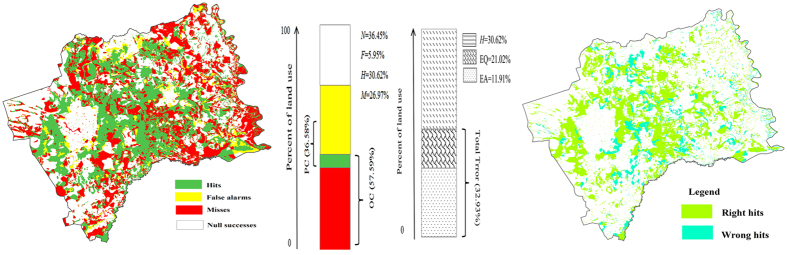
Prediction accuracy and error based on the 1954 (reference), 1932 (reference) and 1932 (simulated) land-use maps (map created using ARCGIS 10).

Figure 10a indicates the percentage of area covered by each land-use category in the reference 1954, reference 1932 and simulated 1932 maps. The largest categories, as indicated by the pie charts, were grassland, arable land and unused land. While the pie charts provide useful information regarding the contribution of each land category, they fail to provide much detail concerning individual transitions among categories. Thus, we overlaid the reference 1954 and 1932 maps, and the reference 1954 and simulated 1932 maps, in order to analyze the changes between 1954 and 1932. Two matrices were produced and are presented in Table 3, and the contribution of each category at the initial time (1954) and at the final time (1932) are indicated. Figure 11b,c and Table 3 illustrate the gross losses and gains in land use types, the majority of which were concentrated in the central parts of the study area as well as in the vicinity of rivers and lakes. The total area change for the reference map was 306,161.00 ha while it was only 195,500.47 ha for the simulated map. Notably, a substantial amount of land classified as unused in 1954 was marked as grassland in 1932 in both the maps, especially the reference map. The spatial location of settlements over time was difficult to reconstruct due to their negligible size compared to the other land-use types, as well as the high temporal variability and plasticity in site preferences^58^. Furthermore, as this research focused on natural factors and excluded other potential drivers (e.g. cultural and socioeconomic driving forces), the reconstruction of settlement areas was unsatisfactory as several differences regarding gross gains and losses existed between the reference and the simulated change maps.

The comparison of observed and predicted change is shown in Fig. 11 where four types of accuracy and error were distinguished. This map was created using an overlay of the predicted land use map of 1932 and the reference map of 1954 in order to reflect landscape persistence versus change. Over the entire study area, the simulated 1932 land use map reported 36.45% null successes, 30.62% hits, 26.97% misses and 5.95% false alarms. Observed change (OC) occurred on 57.59% of the land use, whereas the predicted change (PC) occurred on 36.58% of the land use. Overall, there was a total EQ (error due to quantity) of 21.02%, an EA (error due to allocation) of 11.91% and a total error of 32.93%. This indicates that a minor allocation disagreement and a major quality disagreement existed. The total error is smaller than the observed change, suggesting that this model is more accurate than a null model of no change. The HOC, MOC and FOC ratios were 0.532, 0.468 and 0.103, respectively. The figure of merit (FOM) was 48.19%, which is higher than in some previous case studies^48^,^52^,^53^.

The analysis illustrated that the major differences among the three maps are less concerned with the simulation model; rather, they are related to the discrepancies in land category delineation between 1954 and 1932. The applications with a large FOM are those using the correct, or near-correct, net quantities for the categories in the prediction map. The large number of hits in the HLURM model might be explained by the fact that different definitions exist between the reference map of 1932 and the simulated map of 1932. It is possible that any precise measurement of simulation accuracy is unattainable due to time point inconsistencies concerning the definitions of land use categories. The majority of the topographic maps used in this study were produced between 1932 and 1935 for the Japanese military, while some were produced during the Manchukuo era for the Chinese military. Thus, these maps are limited primarily by the fact that they were created with a specific military purpose in mind. The maps also define certain types of land cover, such as arable land, settlements and water, however, other land-use types, such as unused land, are not clearly reflected. The land cover classes in each map are indicated based on their purpose and criteria. Accordingly, the grassland and unused land types drawn in the 1930 s topographic maps are distinct from those derived from remote sensing images as a result of their different intended uses. For example, grassland is often combined with other land covers in the topographic maps, and thus its boundary is not easily determined. In addition, a large proportion of the grassland in these maps was categorized as wildland, resulting in limited areas of grassland being depicted on the maps. Rainfed lands and blank areas lacking a symbol are often difficult to decipher from grassland and wildland, making it challenging to extract and digitize grassland data. In the context of modern cartographic conventions, unused land is generally under-represented on historical maps. Thus, historical maps possess numerous limitations that must be considered in order to accurately interpret changes in land use. It is also necessary to recognize that discrepancies exist between the topographic maps and remote sensing images in respect to the information depicted. For instance, the period from 1900–1935 has been demonstrated to be a dry period in the history of northeastern China^53^,^59^, confirming that the small area of wetland observed in the maps is an accurate reflection of the land cover of the time. Furthermore, considering the heterogeneity of the data sources, a combination of multi-source data should be used for future research. Blending multi-source data is conducive to extrapolating changes in the environment across a broad range of temporal and spatial scales, resulting in a more reliable representation of land use and land cover change. In comparison with our previous research where a cellular automata Markov model was used to reconstruct the spatial land use patterns in this study area^48^, the HLURM model has performed better due to its consideration of more comprehensive land change driving factors. In this investigation we considered the effects of climate change on land use/cover, and the simulation results were more consistent with historical records. However, artificial adjustment in the quantity control module was only undertaken by considering the climate change drivers in this study. How to incorporate climate data into the simulated model is an area that requires further investigation.

## Conclusions

Historical land use information is essential in understanding how anthropogenic land use and land cover change has influenced the temporal dynamics of environmental and ecological issues. To satisfy the growing demand for harmonized, spatially explicit and high resolution land use change products, a spatial-explicit modeling framework (HLURM: Historical Land Use Reconstruction Model) was explored and proposed in this research to enhance our understanding of the degree and extent of global and regional anthropogenic changes in land use patterns. And then this modeling framework was applied to the historical reconstruction of spatial distribution of land use/cover in the early reclaimed time (1930 s) of Zhenlai County, northeastern China to check its modeling behavior. This was achieved by building a HLURM backward projection model in 90 m × 90 m spatial resolution based on three assumptions: that presently established spatial patterns of land use are intrinsically dependent on the historical patterns; that the boundary of historical land use with human activities does not exceed the union range of each land use type; and that factors relating to land suitability do not change over time. A three-map comparison methodology was then used to validate the projected reconstruction. The main conclusions are:

1. The HLURM model, consisting of four main modules (quantity control, spatial conversion rule, probability and spatial allocation), had a good performance in the spatial reconstruction of various land cover types. A CA model was also used to produce a backward simulation as a carrier.
2. The results of the historical reconstruction revealed that the largest percentage of the study area was grassland, followed by arable land and wetland. The remaining land-use categories comprised relatively smaller areas. Most of the arable land was located in the flat regions in the central and western areas, as well as in the north of the study area. Wetland areas were mainly distributed to the east and south along the Nenjiang and Tao’er Rivers.
3. The total area change in the reference change map between 1954 and 1932 was 306,161.00 ha, while it was 195,500.47 ha for the simulated change map. Gross losses and gains in land-use categories were primarily concentrated in the central areas as well as those areas in the vicinity of rivers and lakes. The expansion of arable land at the expense of grassland was most probably due to the fast population growth experienced over the last few decades. The relative proportion of water bodies increased marginally as a result of increased precipitation. Between 1932 and 1954, a large proportion of grassland was transformed into unused land in both the change maps, especially in the reference change map, indicating that substantial environmental degradation had occurred.
4. The figure of merit of the model was 48.19%, which is higher than that estimated in several other case studies. Error due to allocation was 11.91%, while error due to quantity was 21.01%; this is likely as a result of the inconsistencies concerning category definitions between the maps. The major differences observed among the three maps are less concerned with the simulation model, but are more associated with the inconsistencies regarding how the land-use categories were defined during the study period, especially with regards to grassland and unused land types. For instance, in the topographic maps, grassland is often combined with other land covers, making its boundaries difficult to determine. Furthermore, a substantial portion of grasslands on the maps were often categorized as wildlands, resulted in difficulty extracting and digitizing the spatial extent of the grassland data. Therefore, it is crucial to select a reference map that has achieved high accuracy in the model validation for use in the three-map comparison. Unfortunately, due to the limited availability of historical data, it is often challenging to obtain a suitable reference map to be used to validate a reconstruction model.

## Additional Information

**How to cite this article**: Yang, Y. *et al*. Analyzing historical land use changes using a Historical Land Use Reconstruction Model: a case study in Zhenlai County, northeastern China. *Sci. Rep.* **7**, 41275; doi: 10.1038/srep41275 (2017).

**Publisher's note:** Springer Nature remains neutral with regard to jurisdictional claims in published maps and institutional affiliations.

## Figures and Tables

**Table 1:** Areas and weights of various land use types in the 1930 s.

		Modified data of Markov results	Extractive data from historical documents	Data derived from the topographic maps (DTM)			Weighted area
Land-use categories				i	ii	iii	
Arable land	Area (ha)	146598.14	85938.43	204012.98	143744.76	99902.81	129966.07
	Weight	0.293	0.178	0.089	0.219	0.221	
Forestland	Area (ha)	630.5	—	555.48	881.96	438.98	675.69
	Weight	0.308	—	0.073	0.382	0.237	
Grassland	Area (ha)	221456.34	—	270651.67	346339.5	409115.25	335798.79
	Weight	0.217	—	0.074	0.356	0.353	
Water	Area (ha)	11888.5	—	7755.28	6759.83	6149.02	9274.14
	Weight	0.485	—	0.116	0.254	0.145	
Settlement	Area (ha)	1801.49	—	—	2383.36	5613.1	3758.57
	Weight	0.151	—	—	0.396	0.453	
Wetland	Area (ha)	148313.62	—	48630.73	31496.73	10386.98	51514.18
	Weight	0.202	—	0.157	0.344	0.297	
Other unused land	Area (ha)	916.85	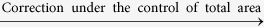				618.69

Note: DTMi - data derived from the topographic maps at scale 1:500,000 in the 1930 s; DTMii - data derived from the topographic maps at scale 1:100,000 in the 1930 s; DTMiii - data derived from the topographic maps at scale 1:50,000 in the 1930 s.

**Table 2:** Factors and their weights used in the construction of suitability maps.

Factors		Arable land	Forestland	Grassland	Water	Settlements	Wetland	Other unused land
Environmental influential factors	Soil	0.232	0.127	0.058	0.294	0.032	0.085	0.021
	Geomorphology	0.054	0.027	0.051	0.209	0.012	0.021	0.063
	Elevation	0.032	0.031	0.031	0.103	0.038	0.143	0.004
	Slope	0.036	0.025	0.071	0.098	0.059	0.111	0.006
human disturbance factors	Aspect	0.005	0.012	0.033	0.071	0.005	0.107	0.009
	Distance from river	0.096		0.099		0.101		
	Distance from roads	0.092	0.072			0.124		
	Distance from settlement	0.213	0.369			0.309		
Spatial auto-correlation factors	Arable land	0.24						
	Forestland		0.337					
	Grassland			0.304			0.162	0.124
	Water				0.225			
	Settlement					0.32		
	Wetland			0.189			0.296	0.205
	Other unused land			0.164			0.075	0.568

**Table 3:** Area counts (ha) of persistence on the main diagonal (underlined) and change from the main diagonal between 1954 and 1932: reference change (in italics) and simulated change (in bold).

		Final Year (1932)						Initial total	Gross loss
				Arable land	Forest	Grassland	Water			Settlement	Unused land
Initial Year (1954)	Arable land	*85202.00*	*122.19*	*76904.66*	*461.23*	*1026.98*	*3638.65*	*167355.71*	*82153.71*
		**120681.57**	**70.78**	**44457.28**	**189.99**	**1547.65**	**408.45**	**167355.71**	**46674.14**
	Forest	*143.27*	*76.74*	*252.40*	*0.00*	*16.24*	*0.00*	*488.65*	*411.91*
		**2.59**	**467.59**	**10.73**	**0.00**	**2.76**	**4.98**	**488.65**	**21.06**
	Grassland	*24937.32*	*534.41*	*123704.28*	*1234.24*	*334.95*	*11626.66*	*162371.86*	*38667.58*
		**1983.44**	**106.57**	**157748.21**	**39.20**	**423.65**	**2070.79**	**162371.86**	**4623.64**
	Water	*3788.96*	*0.00*	*16556.56*	*2797.54*	*64.72*	*2792.23*	*26000.01*	*23202.47*
		**205.99**	**0.00**	**16743.07**	**7517.72**	**3.78**	**1529.45**	**26000.01**	**18482.29**
	Settlement	*1422.64*	*0.00*	*1192.72*	*2.93*	*275.85*	*50.44*	*2944.58*	*2668.73*
		**791.49**	**0.00**	**555.64**	**0.00**	**1584.42**	**13.04**	**2944.58**	**1360.16**
	Un used land	*28250.58*	*148.62*	*127728.90*	*2263.89*	*664.62*	*13388.77*	*172445.37*	*159056.60*
		**6301.00**	**30.76**	**116283.86**	**1527.24**	**196.31**	**48106.16**	**172445.33**	**124339.17**
Initial total		*143744.76*	*881.97*	*346339.52*	*6759.83*	*2383.36*	*31496.74*	*531606.14*	*306161.00*
		**129966.07**	**675.69**	**335798.79**	**9274.14**	**3758.57**	**52132.87**	**531606.14**	**195500.47**
Gross Gain		*58542.77*	*805.23*	*222635.24*	*3962.29*	*2107.51*	*18107.97*	*306161.00*	
		**9284.50**	**208.10**	**178050.58**	**1756.42**	**2174.15**	**4026.71**	**195500.47**	
