# Corrigendum: Analyzing historical land use changes using a Historical Land Use Reconstruction Model: a case study in Zhenlai County, northeastern China

**DOI:** 10.1038/srep45614

**Published:** 2017-04-05

**Authors:** Yuanyuan Yang, Shuwen Zhang, Yansui Liu, Xiaoshi Xing, Alex de Sherbinin

Scientific Reports 7: Article number: 4127510.1038/srep41275; published online: 01 30 2017; updated: 04 05 2017

In the original version of this Article, Affiliation 1 was incomplete. The correct affiliation is listed below.

Institute of Geographic Sciences and Natural Resources Research, Chinese Academy of Sciences, Beijing 100101, China.

These errors have been corrected in both the HTML and PDF versions of the Article.

